# Opening doors for open-source large language models in radiology education

**DOI:** 10.1590/0100-3984.2024.0037

**Published:** 2024-07-11

**Authors:** Partha Pratim Ray

**Affiliations:** Department of Computer Applications, Sikkim University, 6th Mile, PO-Tadong, Gangtok, Sikkim 737102, India. Email: parthapratimray1986@gmail.com.

Dear Editor,

I read with great interest the article by Leitão et al.^(1)^ titled “Performance of ChatGPT on questions from the Brazilian College of Radiology annual resident evaluation test” recently published in **Radiologia Brasileira**. I commend the authors for their insightful and timely study exploring the potential of artificial intelligence (AI), specifically the ChatGPT language model, in the context of radiology education and assessment.

The authors’ methodology of presenting a diverse set of questions from the Brazilian College of Radiology’s annual resident evaluation to ChatGPT and analyzing its performance provides valuable insights into the current capabilities and limitations of such AI systems. The detailed breakdown of ChatGPT’s performance across different question types, topics, and cognitive skill levels helps paint a nuanced picture of where the technology currently stands. I was particularly intrigued by the finding that ChatGPT performed significantly better on questions assessing lower-order cognitive skills and physics-related questions than on those assessing higher-order skills and clinical topics. This suggests that although AI language models like ChatGPT can effectively handle factual recall and basic concepts, they still struggle with the more complex reasoning and domain-specific knowledge required for clinical radiology^(2)^. However, we believe that the potential of open-source large language models (LLMs) extends beyond just ChatGPT, and includes alternatives such as Llama 3, BioGPT, PMC-LLaMA, and BioMistral-7B. These models offer the advantage of being low-cost and free from proprietary dependencies, making them more accessible for radiology education and assessment. As the authors rightly point out, with dedicated training on radiologyspecific content, the performance of these models can be significantly improved^(3,4)^. Beyond just answering questions, such AI systems could be leveraged to generate a wide variety of educational content, including practice questions, explanations, and case discussions, which could serve as valuable study aids for radiology residents. The open-source nature of these models also allows greater flexibility and customization to suit the specific needs of radiology training programs. By exploring and utilizing these alternative open-source language models, the radiology community can tap into the immense potential of AI-assisted education while maintaining control over the technology and avoiding the limitations of proprietary systems like ChatGPT.

The ability of open-source LLMs to provide instant feedback and engage in interactive discussions could revolutionize the way residents learn and practice. Open-source LLMs, which are freely available and can be fine-tuned for specific domains, offer a promising avenue for creating personalized learning experiences in radiology education(5). By integrating these open-source LLMs into radiology curricula, training programs can develop adaptive assessments that cater to the individual strengths and weaknesses of residents, providing targeted feedback and support. In addition, the open nature of these models allows greater collaboration and sharing of resources among radiology educators, enabling the creation of a rich ecosystem of AI-powered educational tools. This could include interactive case studies, virtual mentoring systems, and intelligent study aids that leverage the power of opensource LLMs to provide on-demand support for residents.
